# Severe tumor lysis syndrome during the induction therapy for the treatment of blastic plasmacytoid dendritic cell neoplasm arising from myelodysplastic/myeloproliferative neoplasms

**DOI:** 10.1002/ccr3.3690

**Published:** 2020-12-20

**Authors:** Ken Sagou, Makoto Ito, Yuma Kawamura, Shun Ukai, Miyo Goto, Nobuaki Fukushima, Kazutaka Ozeki, Ryuichi Fukuyama, Akio Kohno

**Affiliations:** ^1^ Department of Hematology and Oncology JA Aichi Konan Kosei Hospital Aichi Japan; ^2^ Department of Hematology and Oncology Nagoya University Graduate School of Medicine Aichi Japan; ^3^ Department of Diagnostic Pathology JA Aichi Konan Kosei Hospital Aichi Japan

**Keywords:** calreticulin mutation, myelodysplastic/myeloproliferative neoplasms, plasmacytoid dendritic cell neoplasm, tumor lysis syndrome

## Abstract

BPDCN shows clinically heterogeneous characteristics. And as other hematological malignancies, symptoms of BPDCN suggesting a high tumor burden, such as high white blood cell count or splenomegaly, should be carefully considered to prevent TLS.

## Graphical abstract

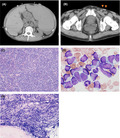

## 1 INTRODUCTION

Blastic plasmacytoid dendritic cell neoplasm is a rare disease with difficulty in diagnosis, and clinical courses of patients with BPDCN vary widely. We report a case of a 63‐year‐old man with BPDCN showing severe tumor lysis syndrome (TLS). BPDCN with high tumor burden should be carefully considered to prevent TLS.

Blastic plasmacytoid dendritic cell neoplasm (BPDCN) is a rare hematological malignancy with dismal prognosis. With difficulty in diagnosis, BPDCN was used in the 2008 World Health Organization (WHO) classification for the first time and became a distinct entity in 2016 WHO classification.^1^ Recently, the presence of four out of five characteristic markers of BPDCN, namely, CD4, CD56, CD123, CD303, and TCL1, has been shown to aid in the accurate diagnosis of BPDCN.^2^ Skin lesions commonly occur in a majority of patients with BPDCN, and blasts of BPDCN are suggested to arise from premalignant hematopoietic precursor clones.^3^ Thus, patients with systemic lesions were treated with conventional chemotherapies for acute lymphoblastic leukemia, acute myeloid leukemia, or non‐Hodgkin’s lymphoma, sometimes followed by autologous or allogeneic hematopoietic cell transplantation as a consolidation therapy.^4^ However, its clinical and biological heterogeneous characteristics make its clinical management difficult.^5^ Herein, we describe the case of a patient who suddenly developed leukemic form of BPDCN accompanied with CALReticulin (*CALR*) mutation arising from myelodysplastic/myeloproliferative neoplasms with ring sideroblasts and thrombocytosis (MDS/MPN‐RS‐T) without cutaneous lesion, showing severe tumor lysis syndrome (TLS) immediately after the induction therapy.

## 2 CASE PRESENTATION

**FIGURE 1:**
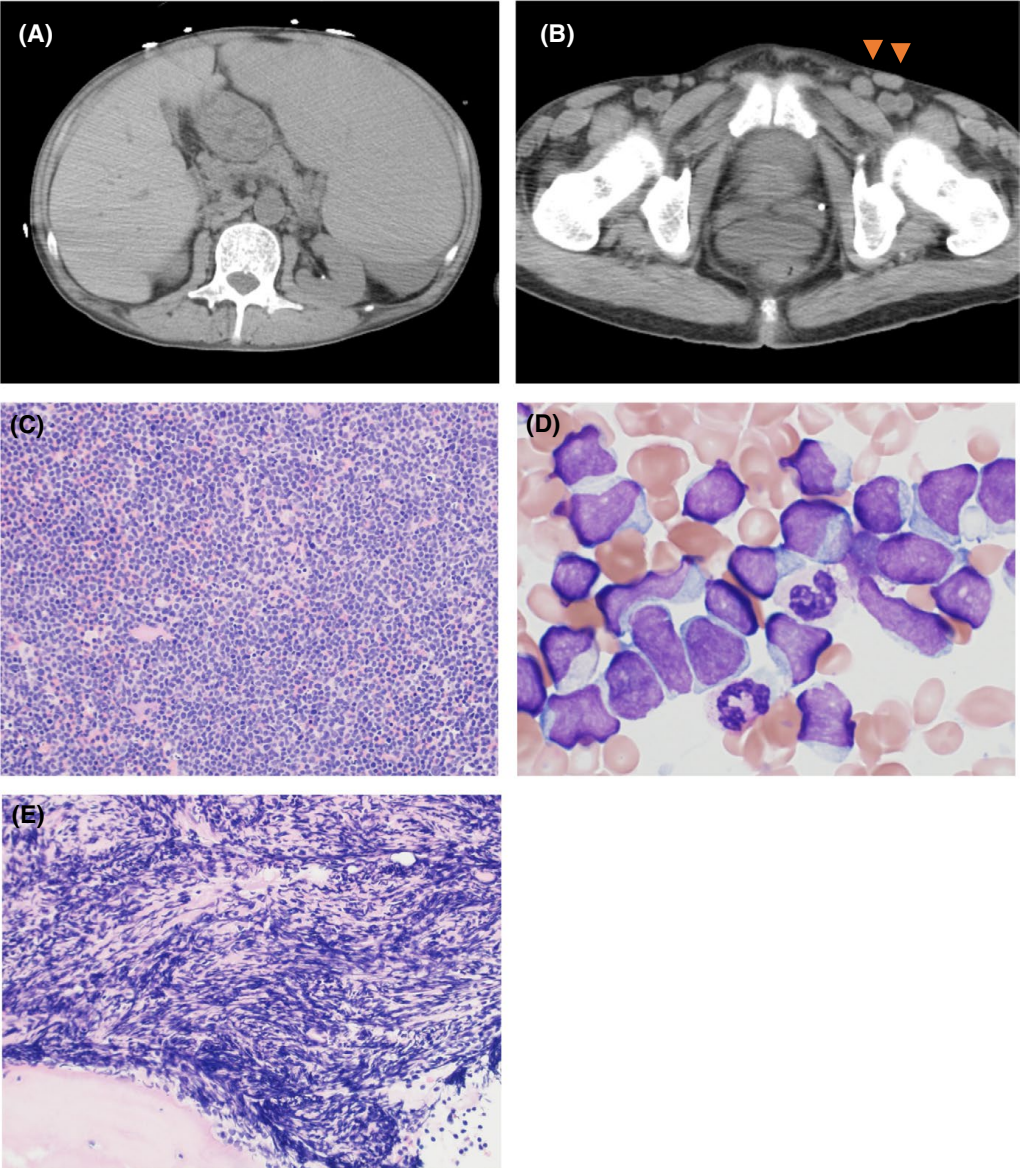
Computed tomography revealed splenomegaly (A) and inguinal lymphadenopathy (B). Markedly hypercellular marrow was occupied by blasts (C), presenting agranular small cytoplasm and fine chromatin (D). Bone marrow biopsy showed severe fibrosis (MF‐3) (E) (magnification of all images ×400).

A 63‐year‐old man previously diagnosed with MDS/MPN‐RS‐T 6 years before the presentation was referred to our hospital for rapid progression of leukocytosis and anemia. Physical examination and computed tomography revealed splenomegaly and inguinal lymphadenopathy without skin involvement (Figure 1 A, B). He did not have fever and had a performance status of 2 according to the Eastern Cooperative Oncology Group score. Blood examination showed the following abnormalities: white blood cell (WBC) count, 44.8 × 10^9^/L with 88% blasts; hemoglobin, 5.5 g/dL; platelet count, 17.0 × 10^9^/L; and serum lactate dehydrogenase (LDH), 300 IU/L. Genetic analyses of his peripheral blood cells detected type 1 *CALR* mutation without Janus kinase 2 mutation. Markedly hypercellular marrow occupied by agranular blasts with small cytoplasm and fine chromatin were detected in the bone marrow aspiration (Figure 1C, D), and bone marrow biopsy showed severe myelofibrosis (MF‐3) (Figure 1E). Flow cytometry performed on bone marrow aspirate revealed that blasts expressed CD4, CD7, CD56, and HLA‐DR without other myeloid and lymphoid markers. Similar blasts occupied the inguinal lymph node, which suggested lymph node involvement. Based on these results, the patient was suspected to have aggressive NK cell leukemia (ANKL), acute leukemia with ambiguous lineage, or BPDCN.

**FIGURE 2:**
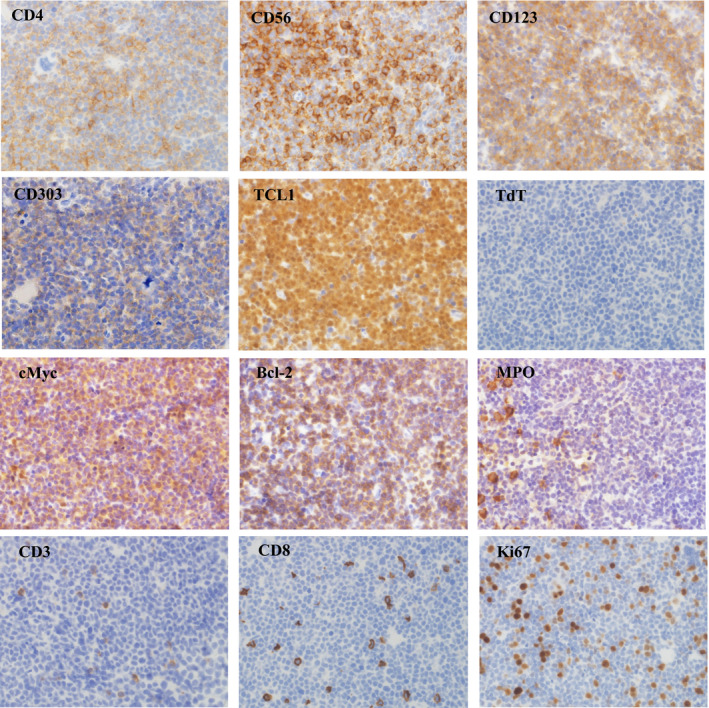
Blasts were positive for CD4, CD56, CD123, CD303, TCL‐1, cMyc, and Bcl‐2 and negative for TdT, myeloperoxidase, CD3, and CD8. Positive rate of Ki67 was approximately 30% (magnification of all images ×400).

Due to rapidly increasing WBC count of 106 × 10^9^/L and worsening malaise, the dose‐reduced SMILE regimen without methotrexate which contained etoposide 70 mg/m^2^ day1–3, ifosfamide 1050 mg/m^2^ day 1‐3, dexamethasone 30 mg/body day 1‐3, and L‐asparaginase 4000 U/m^2^ day 7, 9, 11, 13, 15, 17, and 19 was immediately initiated without accurate diagnosis.^6^ More than 3 L/d of intravenous hydration and 60 mg/d of febuxostat were administered as prophylaxis for tumor lysis syndrome (TLS). Then, immunohistochemistry performed on bone marrow specimen demonstrated that blasts were positive for CD123, TCL‐1, and CD303, and thus, he was diagnosed with BPDCN (Figure 2). In addition, blasts were positive for cMyc and Bcl‐2 and negative for TdT, with relatively low Ki67 index (Figure 2). Cytogenetic analysis revealed normal karyotype of blasts. The urine volume after initiating the chemotherapy was 3000 mL/d, and his condition improved that he could eat a meal the next day. However, his urine volume suddenly decreased, and his condition worsened again on the third day. WBC count immediately decreased; uric acid, potassium, and phosphorus concentrations increased; calcium concentration decreased with rapid increase in serum creatinine concentration, suggesting a clinical TLS (Table 1). Despite the intensive treatment such as aggressive hydration with noradrenaline administration to restore circulation and bicarbonate to correct metabolic acidosis, he finally died of circulatory failure. Autopsy revealed massive ascites and alveolar hemorrhage, whereas only a few blasts remained in bone marrow and spleen.

**TABLE 1:** Blood tests before and after the induction therapy.

		Before treatment	Day 2	Day 3
BUN	mg/dL	34.2	87.9	123.5
Creatinine	mg/dL	1.3	1.6	2.7
UA	mg/dL	6.0	5.5	8.5
P	mg/dL	3.0	17	22.4
Na	mmol/L	137	134	137
K	mmol/L	4.7	7.2	6.9
Ca	mg/dL	7.6	6	6.5
LDH	U/L	395	828	784
WBC	10^9^/L	106	55.7	2.4
Blast	%	96	85	76
RBC	10^12^/L	1.2	1.4	1.1
Hb	g/dL	3.7	4.4	3.3
Platelet	×10^9^/L	17.0	15.0	10.0

Abbreviations: BUN, blood urea nitrogen; Hb, hemoglobin; LDH, lactate dehydrogenase; RBC, red blood cell; UA, uric acid; WBC, white blood cell.

## 3 DISCUSSION

In this case report, the patient was diagnosed with BPDCN arising from MDS/MPN‐RS‐T that progressed into clinical TLS immediately after the initiation of chemotherapy. Majority of patients with MDS/MPN‐RS‐T have a *SF3B1* mutation.^7^ Although *SF3B1* mutations were not examined in this case, morphologically apparent dysplasia and the presence of ringed sideroblasts strongly supported the diagnosis of MDS/MPN‐RS‐T. On the other hand, while there is one case report about a patient with MDS/MPN‐RS‐T that was positive for both *CALR* and *SF3B1* gene mutations,^8^ BPDCN with *CALR* mutation has not yet been reported to the best of our knowledge. Therefore, in this case, MDS/MPN‐RS‐T might already have been accompanied with *CALR* mutation.

TdT negativity was reportedly associated with inferior survival in BPDCN;^9^ however, whether the aggressive clinical course observed in this case was typical for TdT‐negative BPDCN remains unknown. Conversely, majority of patients with BPDCN expressing cMyc were reportedly accompanied by 8q24 rearrangement, immunoblastoid morphology, and skin lesions.^10^ Although 8q24 rearrangement was not examined in this case, the patient had no skin lesions and his blasts showed classic cytomorphology, which suggested that his clinical features were not typical for MYC‐positive BPDCN.

Previous studies showed that BPDCN with leukemic presentation without skin manifestation rarely occurs and tends to present with cytopenia rather than leukocytosis.^11^ However, although the patient had leukemia at diagnosis, it was not accompanied with skin lesions and showed very high WBC count, suggesting it to be a very rare case. Moreover, rapid increase in WBC counts and massive splenomegaly indicated a very high tumor burden and rapid tumor growth. The SMILE regimen was selected because it was effective for ANKL^12^ and the etoposide contained in SMILE regimen was also effective for BPDCN.^13^ Although occurrence of TLS was cautiously considered in spite of low Ki67 index and enough hydration and febuxostat were provided to the patient for the prevention of TLS, he developed fatal TLS. More rigorous management including the early use of rasburicase and induction of hemodialysis is required.

Although the risk prediction of TLS in BPDCN is unknown because of the rarity and heterogeneity of this disease, the high‐disease burden may indicate the risk of TLS as same as other hematological malignancies, and the evaluation of peripheral blood WBC counts and serum LDH concentration may be useful for risk classification of TLS in BPDCN as in acute leukemia.^14^

In conclusion, BPDCN shows clinically heterogeneous characteristics, and patients with clinical symptoms suggesting an aggressive clinical course with high tumor burden, including high WBC count or splenomegaly, should be carefully considered to prevent TLS.

## CONFLICT OF INTEREST

There are no conflicts of interest to report.

## AUTHOR CONTRIBUTIONS

KS: selected the patient and designed the manuscript. KS, MI, NF, and AK: wrote the manuscript. RF: performed the immunohistochemistry. All the authors: reviewed the paper and agreed with the final version.

## ETHICAL APPROVAL

This study was approved by the institutional ethical committee of the Konan Kosei Hospital. Written informed consent was obtained from the family of the patient for publication of this case report.

## Data Availability

The data that support the findings of this study are available on request from the corresponding author. The data are not publicly available due to privacy or ethical restrictions.
